# Optimized S-Curve Transformation and Wavelets-Based Fusion for Contrast Elevation of Breast Tomograms and Mammograms

**DOI:** 10.3390/diagnostics13030410

**Published:** 2023-01-23

**Authors:** Vikrant Bhateja, Shabana Urooj, Anushka Dikshit, Ashruti Rai

**Affiliations:** 1 Department of Electronics Engineering, Veer Bahadur Singh Purvanchal University, Shahganj Road, Jaunpur 222003, Uttar Pradesh, India; 2 Department of Electronics and Communication Engineering, Shri Ramswaroop Memorial College of Engineering and Management, Faizabad Road, Lucknow 226028, Uttar Pradesh, India; 3 Department of Electrical Engineering, College of Engineering, Princess Nourah bint Abdulrahman University, P.O. Box 84428, Riyadh 11671, Saudi Arabia; 4 Dr. A.P.J. Abdul Kalam Technical University (AKTU), Lucknow 226031, Uttar Pradesh, India

**Keywords:** Absolute Mean Brightness Error (AMBE), breast tomographic images, contrast enhancement, DWT, fusion metrics, PSO and S-curve transform

## Abstract

For the purpose of accuracy in detection and diagnosis, Computer-Aided Diagnosis (CAD) is preferred by radiologists for the analysis of Breast Cancer. However, the presence of noise, artifacts, and poor contrast in breast images during acquisition highlights the need for sophisticated enhancement techniques for the proper visualization of region-of-interest (ROI). In this work, contrast elevation of breast mammographic and tomographic images is performed with an improved S-Curve transform using the Particle Swarm Optimization (PSO) algorithm. The enhanced images are assessed using dedicated quality metrics such as the Enhancement Measure (EME) and Absolute Mean Brightness Error (AMBE) measurement. Although the enhancement techniques help in attaining better images, certain features relevant for diagnosis purposes are removed during the enhancement process, creating contradictions for radiological interpretation. Hence, to ensure the retention of diagnostic features from original breast tomograms and mammograms, a Discrete Wavelet Transform (DWT)-based fusion approach is incorporated, which fuses the original and contrast-enhanced images (with optimized s-curve transformation function) using the maximum fusion rule. The fusion performance is thereafter measured using the Image Quality Index (IQI), Standard Deviation (SD), and Entropy (E) as fusion metrics.

## 1. Introduction

Breast cancer is the second most deadly cancer among women across the globe, as evident from recent statistics: 2.3 million new cases are estimated worldwide [1]. With technological advancements, various modalities are available for the purpose of detection and diagnosis of Breast Cancer. Various factors such as age, the stage of cancer, breast tissue density, etc., are responsible for the efficacy of these modalities such as X-rays, MRIs, and so on. Out of these modalities, mammography and tomography are considered to be the most reliable tools for the screening of Breast Cancer [2,3]. However, concerning the image quality, there is a need for high-level expertise for recording accurate analysis and measurements for radiological interpretation via Computer-Aided Diagnosis (CAD) [4]. Contrast enhancement techniques are applied for visual quality elevation to improve the processes involved in CAD. Histogram Equalization is the most commonly used enhancement technique but results in a highly overbright image, which is unsuitable for medical images such as mammograms. The Bi-dimensional Empirical Mode of Decomposition (BEMD) maps values from the input gray-scale to the output gray-scale values using intrinsic mode functions [5]. Local Entropy Maximization Histogram Equalization and DWT-based image Decomposition [6] are used to overcome the drawbacks of Histogram Equalization. Contrast-Limited Adaptive Histogram Equalization (CLAHE) has become rather popular, resulting in better contrast and easing the detection of highly minute microcalcifications in dense breast tissues, but has highlighted the creation of artificial boundaries in the images, creating contradictions in analyzing the medical images in general [7]. An improved version of the CLAHE algorithm applies the double sigmoidal function, which is implemented by dividing the image into blocks rather than entirely on the image [8,9,10]. In order to optimize the enhancement techniques for compatibility with different types of databases, an intensive study has been carried out on the nature-based approaches such as Ant Colony Optimization (ACO) and Firefly and Particle Swarm Optimization (PSO) because of their ability to provide optimal solutions to a variety of functions [11,12,13,14,15,16]. A large study has been carried out on the wavelets-based fusion approach and Hilbert transform. In wavelet approaches, SWT- and DWT-based fusion approaches have provided desirable results for breast cancer diagnosis, out of which DWT has shown better performances. Upon attaining the enhanced breast image as the output, desired image features are extracted from them to understand the nature of the tumor and classify it accordingly for relevant diagnosis [17,18,19,20,21,22,23].

However, it is noteworthy that during the course of improving the visual quality of the breast image, the contrast-enhancement techniques tend to remove many important features from the image, thereby limiting the amount of information available for the detection of the tumors at an early stage [24,25,26,27,28,29]. This paper proposes an improved approach for this purpose in order to elevate the radiological interpretation of breast tomogram and mammogram images. To overcome the aforesaid ailments of the enhancement approach, the optimization of the S-Curve transform is carried out by modifying the sigmoidal function and its tuning parameters with the help of the PSO algorithm. To further support the loss of important features during the enhancement, the concept of image fusion has been deployed, which fuses the images unimodally, i.e., images belonging to the same modality (original and enhanced breast images). The paper is organized into the following sections: Section 1 provides an introduction to the subject along with a review of related works. Section 2 details the Optimized S-Curve Transform for Enhancement followed by a discussion of the proposed Wavelet-based fusion approach in Section 3. In Section 4, the analysis and discussion of the obtained results are presented along with the performance evaluation measures. Section 5 concludes the paper.

## 2. Optimized S-Curve Transform for Contrast Enhancement of Breast Images

The motive of enhancement techniques is to eradicate the numerous hurdles that hamper the radiological diagnosis such as blurred edges, low contrast, undesirably high contrast, and so on. Thus, the S-Curve transform aims to overcome these hurdles by providing precise management of the image pixels necessary for the quantitative and qualitative assessment. It refers to an extended version of the gray-level transformation resulting in a sigmoid function-like curve [7]. The application of this technique directly on a given image may lead to over-brightening as a result of the brightening of regions of the least importance for radiological interpretation. The S-Curve performs the contrast elevation by increasing the difference between the low- and high-frequency components of the image in such a way that the intensities of the pixels are preserved, and this is referred to as the global S-Curve transform. However, the incrementation in this transformation becomes undesirably high. Thus, to resolve this, the S-Curve operation is applied locally by dividing the input image into kXk blocks/tiles. The contrast after applying this method is improved without creating artificial boundaries. The local S-Curve transform used for the proposed approach of enhancement is given by Equation (1).

(1) $$S=\alpha +\left[\frac{\left(\beta -\alpha \right)}{1+e^{-\frac{\left(r-\gamma \right)}{\delta }}}\right]$$

where *α*, *β*, *γ*, and *δ* are constants set as:

$$\alpha =0.9642.\beta =8.594*10^{-4}.\gamma =0.4962.\delta =0.07598$$

based on the simulation results and tuning of parameters; *r* is the value of a normalized current pixel in the range of [0,1] and *s* is the value of the transformed pixel obtained in the range of [0,1], which is again de-normalized into the range [*Lmin*, *Lmax*] as per the requirement of the S-Curve Transform [8].

The implementation of Equation (1) on medical images provides an adequate difference between the intensities of high and low pixels giving effective contrast enhancement. However, the values of the coefficients *α*, *β*, *γ*, *δ*, and *r* are subject to change based on the nature/type of mammogram or tomogram during simulation. Thus, while dealing with a database containing multiple images, the values require adjustments for every image, which, if performed manually, is a very tedious task. Therefore, there is a need for an automated approach, which can provide the best values for these coefficients so as to improve the contrast of the tomographic and mammogram images.

The optimization of the enhancement function of Equation (1) is carried out using the PSO algorithm. The algorithm comprises a group of birds or swarms referred to as the solution, which moves across the search space, which is essentially the dimension of the input image taken into consideration. The first step to implementing this algorithm is to initialize the solution and define the dimensions of the search space, i.e., the image. The solution is then made to move across the image and update it after every iteration in terms of position and velocity [14]. The update of the particles on every iteration is performed when the particles perform the following search in terms of finding the best solution of the fitness function, while the second-best solution is still searched for by the particles.

The position of the particle is updated using Equation (2).

(2) $$xi^{t+1}=xi^{t}+vi^{t}\times t$$

where *xi*^*t*+1^ is the updated position and *xi*^*t*^ is the current position of the particle. Similarly, the velocity of the particle is updated using Equation (3).

(3) $$v^{i}{}_{k+1}=wv_{k}{}^{i}+\left(c_{1}r_{1}xBest^{t}{}_{i}-x^{t}{}_{i}\right)+\left(c_{2}r_{2}gBest^{t}{}_{i}-x^{t}{}_{i}\right)$$

where *xBest* is the best particle solution, *gBest* is the best group position, *w* is the inertia weight, *𝑐*_1_ and *𝑐*_2_ are the two positive constants, and *𝑟*_1_ and *𝑟*_2_ are two random parameters within [0,1] [15].

Thus, the position is updated after every update using the expression given in Equation (4).

(4) Present Position=Current Position=Velocity(v)

Another aspect of optimization is the fitness function in which, if its value is greater than the value of *gBest*, then it is updated as the current fitness function, which uses EME as one of the parameters. EME measures the degree of contrast enhancement by dividing the image into non-overlapping blocks (𝑏_1_ × 𝑏_2_) with the dimensions of 3 × 3 and averaging the values of the maximum and minimum intensity *(I_max_* and *I_min_).* It is expressed as:

(5) $$EME=\frac{1}{b_{1}b_{2}}\overset{b_{2}}{\underset{j=1}{\sum }}\overset{b_{1}}{\underset{i=1}{\sum }}20\log\frac{I_{max}}{I_{min}}$$

The proposed optimized enhancement approach can be summarized with the help of Algorithm 1 given below:
**Algorithm 1:** Procedural Steps for Optimized S-Curve Transform for Contrast Enhancement***   BEGIN*****Step 1:***Input* test mammogram/tomogram image.**Step 2:***Convert* from RGB to Grayscale.**Step 3:***Apply* S-Curve transform using Equation (2).**Step 4:***Initialize* the variables for PSO algorithm.**Step 5:***Define* the objective function and fitness function.**Step 6:***Compare* the values of *xBest* and *gBest* for every iteration.**Step 7:***Update* the position and velocity of the particle.**Step 8:***Repeat* steps 4 to 6 for all iterations until best value of EME is obtained.**Step 9:***Output* images are obtained by substituting values in Equation (2).***END***

## 3. Proposed Wavelet-based Medical Fusion Approach for Breast Images

Contrast-enhancement techniques tend to elevate the overall contrast of the images by focusing on the regions of prime importance. However, it has been observed in most cases that a loss of relevant features occurs, which are essential for radiologist interpretation, as a result of being eradicated as noise in most of the enhancement techniques [21]. Therefore, in order to restore these relevant features, the original and enhanced images are fused together, resulting in an output-fused tomographic or mammographic image, which contains the features of both the original and enhanced images. Although most of the fusion modalities are multimodal in nature combining images of different modalities together, the proposed Wavelet-based Medical Image Fusion Approach aims to perform image fusion for mammogram and tomographic images, making it unique from the other state-of-the-art fusion approaches.

### 3.1. Discrete Wavelet Transform

Wavelets are essentially mathematical solutions that are used to satisfy a considered mathematical function or simply represent a mathematical function or data. This is achieved by splitting an image into different frequency components and analyzing it in reference to a given scale or window. The advantage of using a wavelet transform is that it ensures immediate access to the required information, which may be overlooked in various time–frequency transforms such as the Fourier transform. The Discrete Wavelet Transform (DWT) is similar to the concept of filtering a particular non-overlapping bandwidth from multiple banks of filters varying by an octave. The process is quite easy to implement by using wavelets and effectively reduces the calculation time and resources required. The Inverse Discrete Wavelet Transform (IDWT) is the reverse of the DWT, which is responsible for the reconstruction of the coefficients created during the DWT back into the image. This is performed by upsampling by inserting zeros into the samples followed by a filtering technique to expand the signal [8,19].

### 3.2. Maximum Fusion Rule

The Maximum Fusion rule is based on the selection of only those pixels of the image taken into consideration that have the maximum intensity amongst the other pixels of the entire image and is given by the formula below.

(6) $$F\left(i,j\right)=\overset{m}{\underset{i=0}{\sum }}\overset{n}{\underset{j=o}{\sum }}max\;A\left(i,j\right)\;B\left(i,j\right)$$

where *F*(*i*,*j*) is the output image and *A*(*i*,*j*) and *B*(*i*,*j*) are the input images. Amongst the other fusion rules, the maximum fusion rule seems to be a better choice for the proposed fusion approach as it helps in concentrating on the region where the tumor is located in the breast tissues in the mammograms/tomograms [18].

### 3.3. Proposed Fusion Scheme

**Figure 1:**
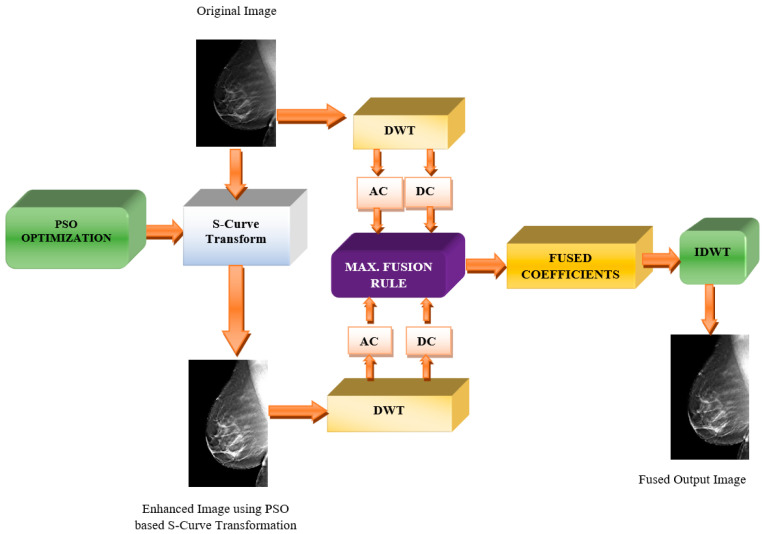
Block Diagram Showing Proposed Wavelet-based Medical Fusion Approach for Breast Images.

The fusion of the mammogram or tomogram images proposed in the paper is carried out using DWT-based fusion using the maximum fusion rule. The purpose of fusion in breast images is to restore the possibly lost important features for radiological interpretation. As illustrated in Figure 1, the source image (which can be mammographic or tomographic images) is taken from an input dataset. The original image is then enhanced using the optimized S-Curve Transform via optimal tuning of the transformation function parameters. It is known that DWT operation is performed on both images individually by utilizing the properties of the high-pass and low-pass filters to decompose the image into the coefficients. Hence, DWT is applied to both the original and enhanced medical images resulting in frequency components from both images, which are the low-frequency Approximate Coefficients (AC) and high-frequency Detailed Coefficients (DC). The fusion will take place between the AC and DC of both the original and enhanced images, respectively, using the Maximum Fusion rule. Lastly, IDWT is performed upon obtaining the resultant fused AC and DC to yield the final fused output image.

## 4. Experimental Results and Discussions

### 4.1. Image Quality Assessment (IQA)

The enhancement of an image is fundamentally the modification of the pixel values. However, due to the amplification of the noise and the overbrightness in the image, one cannot rely solely on the contrast improvement to assess the visual quality of an enhanced mammogram or tomogram. The evaluation is therefore performed using dedicated IQA metrics such as EME and AMBE [5]. For performance evaluation of the fusion approach, the fusion metrics deployed are Entropy (E), Standard Deviation (SD), and IQI [6,7,30,31,32].

### 4.2. Experimental Set-Up

The test images used for simulations in this paper are taken from three distinct benchmarking databases: (a) The Mini-MIAS database of the Mammographic Image Analysis Society (MIAS), University of Cambridge, UK [33]. It is a repository containing 322 digitalized mammograms reduced to a 200-micron pixel edge with the dimensions 1024 × 1024; (b) The Curated Breast Imaging Subset of Digital Database of Screening Mammography (CBIS-DDSM) [34]; (c) the Digital Breast Tomosynthesis (DBT) Database, which presents a three-dimensional view of mammograms procured from the CAD-DBT system in Torino, Italy, which contains the DBT mammograms of 175 patients in JPG format with the dimensions 425 × 226 × 8 [35]. These datasets include normal, actionable, biopsy-proven benign and cancerous cases in both craniocaudal (CC) and mediolateral (MLO) views. The input images are pre-processed by converting them from RGB to gray-scale format and then normalized to bring the pixels to the range of [0 1]. The gray-scale images are subjected to the application of the s-curve transformation function; their respective parameters are optimized using the PSO algorithm. During the implementation of PSO, Swarm Size is 30 and the Number of iterations is 10. The α and β parameters of Equation (1) are tuned with PSO, while *r*, *γ*, and *δ* are treated as constants; *r* is the value of the normalized current pixel in the range of [0,1] and lambda = 0.4962.

### 4.3. Enhancement Response of PSO-Optimized S-Curve Transformation

**Figure 2:**
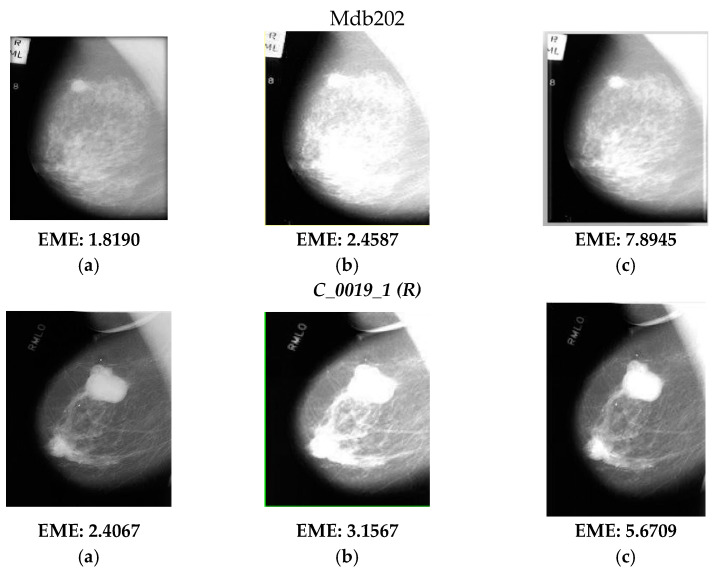

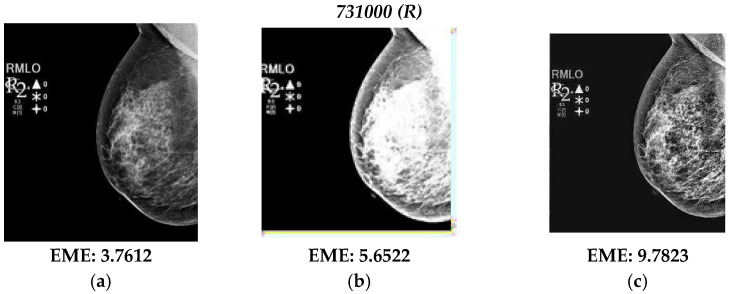
(**a**) Original Breast Images (Mammograms/tomograms); enhancement results obtained using (**b**) S-curve Transformation Function; (**c**) Optimized S-curve Transformation Function (Proposed).

The visual results in Figure 2 show three cases; the first two are mammograms and the last one is a tomogram. It is evident in Figure 2b that the visual response by enhancement using the s-curve transformation function of Equation (1) yields medical images with overbrightness, causing saturation of gray-levels leading to a lack of visibility in certain regions, thereby obscuring the requisite diagnostic features. Although it could be demonstrated via Figure 2c that the response of enhancement is improved to a significant extent with the optimized s-curve transformation function. This is achieved by incorporating the PSO algorithm into the existing s-curve transform; the α and β parameters of Equation (1) are also tuned to their optimal values to yield maximum values of EME. The same can be illustrated by the increasing values of EME as shown for all three test cases in Figure 2.

### 4.4. Fusion Response and Performance Measurements

**Figure 3:**
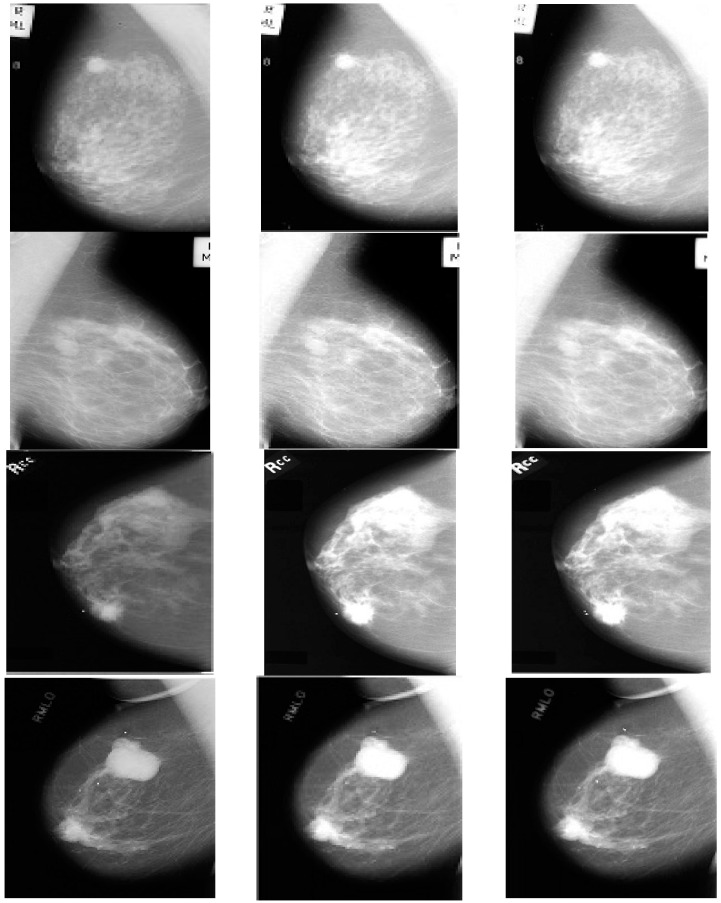

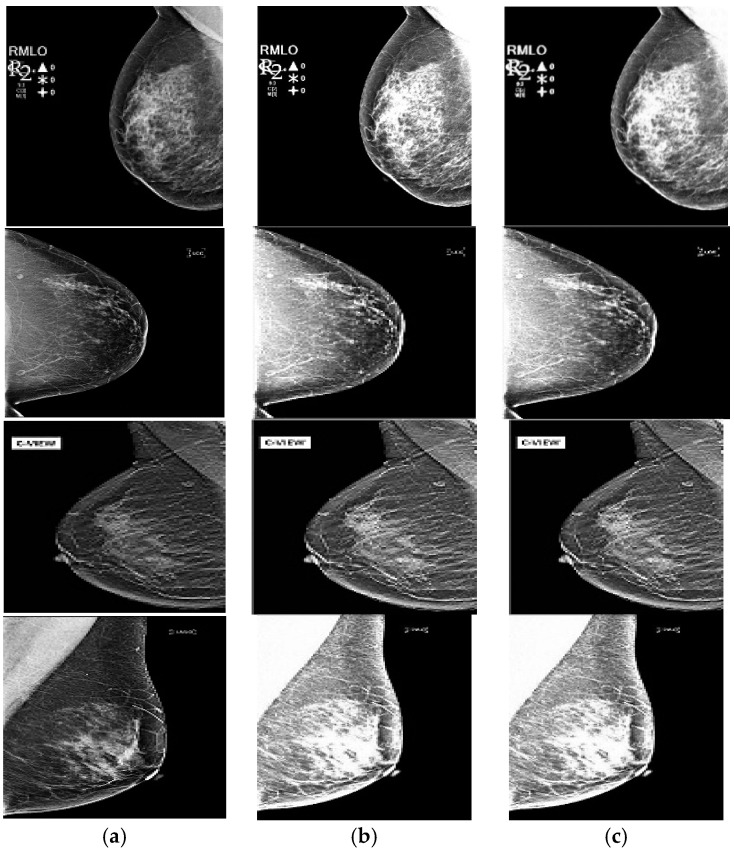
(**a**) Original images from MIAS, DDSM, and DBT Databases, (**b**) Enhanced Images obtained via Optimized S-curve Transformation, (**c**) Fused Images.

The original source images and enhanced images are then processed via sub-band decomposition using DWT via the suitable wavelet family followed by fusion using the max-max fusion rule. The obtained results are shown in Figure 3, followed by performance measurement using various IQA metrics/fusion metrics and enlisted in Table 1 and Table 2.

As already mentioned, the IQA of test images was carried out using E, EME, and AMBE to quantify the enhancement response, and SD and IQI metrics quantify the fusion response for the original and enhanced images shown in Figure 2 and Figure 3, and their values are tabulated in Table 1 and Table 2, respectively. As per the tabulated results in both tables, it can be observed that the incremental values of quality metrics are seen for the fused image of Figure 3c with respect to the source images in Figure 3a,b. The improvement in values of Entropy and Standard Deviation shows that the fused images have better information restoration and redundancy minimization with respect to the source images. Further, betterment in terms of EME and AMBE depicts that the fusion process has maintained the contrast and brightness of the medical images leading to proper visualization of ROI without the introduction of any other artifacts. The sharpness of the fused image is also improved as shown by enhancing values of IQI.

### 4.5. Discussions

The above section of results shows the incremental values of the sourced mammogram and tomographic images after being enhanced using Particle swarm optimization and Image fusion. For instance, in the case of the source test images from the MIAS Dataset (mdb202rl, mdb019rm, mdb145lx, and mdb099xy), it can be seen that the original sourced images have very low values of Entropy (E) referring to the amount of information content present in the input images. This is evidence that these images have low resolution and poor contrast during acquisition. EME and SD denote the measure of contrast enhancement in the source and output images, whereas IQI tends to assess the degree of sharpness. Upon performing the optimized S-Curve transform on the test images, there is a significant increase in the values of EME and E as demonstrated in Table 1. It can be seen in the enhanced images that the Region of Interest (ROI) has been substantially improved, visualizing the breast abnormality for further investigation.

Fusion of original and enhanced images was carried out to make up for the loss of features and introduced artifacts during the enhancement process in the breast images. Upon performing the fusion, the IQA values have shown an improvement such as in the case of mdb202rl in which the value of entropy increased from 5.8901 to 6.8923 and the value of EME increased from 1.8190 to 11.4356, which is evident when viewing the image visually. For the tomographic images sourced from CAD-DBT for image Case9_7110000_R_CC, the values of the original Entropy and EME are 4.4013 and 2.5643, which, upon enhancement increased, to 6.7623 and 9.9023, and upon performing image fusion, it increased further to 7.7634 and 12.0933. Hence, it can be seen that the proposed approach for enhancement can be performed on both the mammogram and tomographic images. The response of SD values in Table 2 demonstrated that the contrast is not disturbed during fusion, and the redundancy was also reduced. Further, the incremental values of IQI show that the sharpness is well retained. These results are far better than the conventional approaches of Histogram Equalization (HE), Adaptive Histogram Equalization (AHE), and Contrast-Limited Adaptive Histogram Equalization (CLAHE) as shown in the response in Figure 2b wherein the EME values are low in comparison to those of Figure 2c. It is noteworthy that the conventional s-curve transformation is the basic working transform function of HE, AHE, and CLAHE approaches with minor variations. Hence, this could indicate that enhancement using optimized s-curve transformation has shown an improvement over traditional HE-based approaches (Conventional CAD).

## 5. Conclusions

In this paper, PSO-based optimization of the s-curve transformation function was carried out for breast mammograms and tomograms. The optimized tuning of parameters of the s-curve transfer function using the PSO algorithm improved the brightness and contrast of the breast images leading to proper visualization of abnormalities. In order to maintain proper contrast and retain other diagnostic features, the fusion of the original and enhanced breast images is carried out using a combination of DWT and the max-max fusion rule. The proposed PSO-based optimization improves the overall contrast of the ROI and the retention of diagnostic features and minimizes redundancy. This process leads to an elevation of the overall accuracy of the CAD system for breast cancer. Fusion performance and contrast enhancement have been validated with the measurement of different quality metrics such as E, EME, AMBE, SD, and IQI. The proposed approach succeeds in providing visually improved breast images for radiologists to consider as a second opinion, making it possible not only to detect the presence of the tumor but also to monitor the stage of Breast Cancer.

## Figures and Tables

**Table 1:** Quantification of Enhancement Response using E, EME, and AMBE.

Sample Images	Original (E)	Enhanced (E)	Fused (E)	Original (EME)	Enhanced (EME)	Fused (EME)	Fused (AMBE)
mdb202rl.jpg	5.8901	7.2250	6.8923	1.8190	7.8945	11.4356	27.6414
mdb019rm.jpg	7.9949	7.8785	7.7823	2.5691	8.9834	13.6798	34.6685
mdb145lx.jpg	5.4030	6.8701	8.893	1.9655	4.6723	15.7813	27.6714
mdb099xy.jpg	2.1567	3.4509	3.4509	3.5612	7.7623	12.6509	36.4454
C_0006_1.Right	6.2634	7.5609	7.9987	2.1841	9.7823	12.6750	44.9799
C_0009_1.Right_CC	7.9949	8.1234	8.9945	2.5691	3.6723	17.6799	33.0357
C_0019_1.Right_MLO	6.1792	7.5478	8.2672	2.4067	5.6709	11.6430	16.7191
D_4084_1.Right_CC	6.5118	8.7801	8.9302	2.9893	4.7802	15.7801	20.4578
Case2_7210000_L	6.2634	7.5609	8.8812	2.1841	9.7823	16.7543	27.4422
Case9_7110000_R_CC	4.4013	6.7623	7.7634	2.5643	9.9023	12.0933	27.5516
Case13_Series002.jpg	4.0759	5.8907	6.9015	5.1510	5.7801	10.7813	16.9724
Case42_Series098.jpg	5.4378	6.8956	7.0091	3.7612	7.3467	14.9025	23.6698

**Table 2:** Quantification of Fusion Response using IQI and SD.

Sample Images	Original (IQI)	Enhanced (IQI)	Fused (IQI)	Original (SD)	Enhanced (SD)	Fused (SD)
mdb202rl.jpg	0.5887	0.6799	0.6932	87.6701	91.7765	98.0106
mdb019rm.jpg	0.7891	0.7234	0.7881	88.7812	84.8903	95.4732
mdb145lx.jpg	0.4801	0.6720	0.6917	78.9024	85.8976	95.1043
mdb099xy.jpg	0.6578	0.6332	0.7230	77.7834	91.8902	96.7891
C_0006_1.Right	0.4501	0.5632	0.7834	90.6712	76.8934	99.6734
C_0009_1.Right_CC	0.4532	0.5667	0.6698	89.1234	85.8044	94.9044
C_0019_1.Right_MLO	0.6990	0.7623	0.7893	72.8901	78.9032	98.6712
D_4084_1.Right_CC	0.6722	0.7721	0.8894	91.8903	87.7823	95.4989
Case2_7210000_L	0.6398	0.6923	0.7834	78.4530	88.9045	98.5623
Case9_7110000_R_CC	0.7892	0.8091	0.9854	88.7834	81.2345	93.6745
Case13_Series002.jpg	0.8936	0.8792	0.9367	89.9034	80.9038	96.8940
Case42_Series098.jpg	0.5906	0.7823	0.7804	81.8923	87.8933	98.7831
